# Visual attention spreads broadly but selects information locally

**DOI:** 10.1038/srep35513

**Published:** 2016-10-19

**Authors:** Satoshi Shioiri, Hajime Honjyo, Yoshiyuki Kashiwase, Kazumichi Matsumiya, Ichiro Kuriki

**Affiliations:** 1 Research Institute of Electrical Communication, Tohoku University.

## Abstract

Visual attention spreads over a range around the focus as the spotlight metaphor describes. Spatial spread of attentional enhancement and local selection/inhibition are crucial factors determining the profile of the spatial attention. Enhancement and ignorance/suppression are opposite effects of attention, and appeared to be mutually exclusive. Yet, no unified view of the factors has been provided despite their necessity for understanding the functions of spatial attention. This report provides electroencephalographic and behavioral evidence for the attentional spread at an early stage and selection/inhibition at a later stage of visual processing. Steady state visual evoked potential showed broad spatial tuning whereas the P3 component of the event related potential showed local selection or inhibition of the adjacent areas. Based on these results, we propose a two-stage model of spatial attention with broad spread at an early stage and local selection at a later stage.

Spatial spread is a critical aspect of visual attention. The focal point of attention does not provide information of the extent of interest, which determines the part of an image attended (e.g., the nose, configuration of individual face parts, the whole face or the entire upper body, when a viewer looks at the center of a face). There are two factors that must be considered for understanding the spatial extent of attention, namely, spread of enhancement (spread for short) and inhibition. Both spatial spread and inhibition around the attentional focus have been reported: spread^1^,^2^,^3^,^4^,^5^,^6^,^7^ and inhibition^8^,^9^,^10^,^11^,^12^,^13^ around the attentional focus. It is still an open question whether spread and inhibition are controlled by a single flexible attentional process that changes the spatial profile via selective inhibition depending on task requirements and conditions or by two distinct processes with different spatial properties. Multistage models of attention^14^,^15^,^16^ are consistent with the two-process interpretation, but there is no report of differences in spatial property at different stages of attention processing.

This report shows electroencephalogram (EEG) evidence for the attentional spread and selection/inhibition being at different stages of visual processing. We estimated attentional modulation in space by steady state visual evoked potential (SSVEP)^17^,^18^,^19^,^20^,^21^,^22^,^23^ and by event related potential (ERP)^24^,^25^,^26^. Analyzing the same EEG signals of the same participants obtained in the same attention experiment, the SSVEP showed broad spatial tuning whereas the P3 component of the ERP showed narrow spatial tuning, which can be attributed to difference in spatial attention at early and late stages of visual processing. Behavioral results also support this view, showing differences in spatial attention for different tasks. A selective response was found at the attentional focus for the task to detect a target letter among distractor letters whereas spread into a larger area was found for the task to detect luminance decrements in a dual task experiment, where the target letter detection and luminance decrement detection are asked in the same trials.

We estimated the modulation of spatial attention on EEG signals while participants were performing a rapid serial visual presentation (RSVP) task at one or two of eight locations (Single and Double cue conditions). Stimuli were disks arranged circularly around the fixation point that participants were instructed to fixate on throughout a trial (Fig. 1(a)). The Double cue condition was designed to show ignorance/suppression between the two cued locations. In the analysis, we first extracted SSVEP components associated with each disk location. SSVEPs are oscillatory EEG potentials that occur synchronously with flickering visual stimuli, and the amplitude of each corresponding temporal frequency is modulated by attention to each flickering stimulus. The SSVEP amplitude of each temporal frequency as a function of distance from the attention focus should provide information on the spatial modulation of attention. We also analyzed inter-trial phase coherence (ITPC), an index of phase coherence in neural activity calculated from phase variation of the flicker frequency components, across trials. A higher ITPC indicates a greater degree of phase coherence and larger attentional modulation^18^,^19^,^27^,^28^,^29^.

## Results

**Figure 1:**
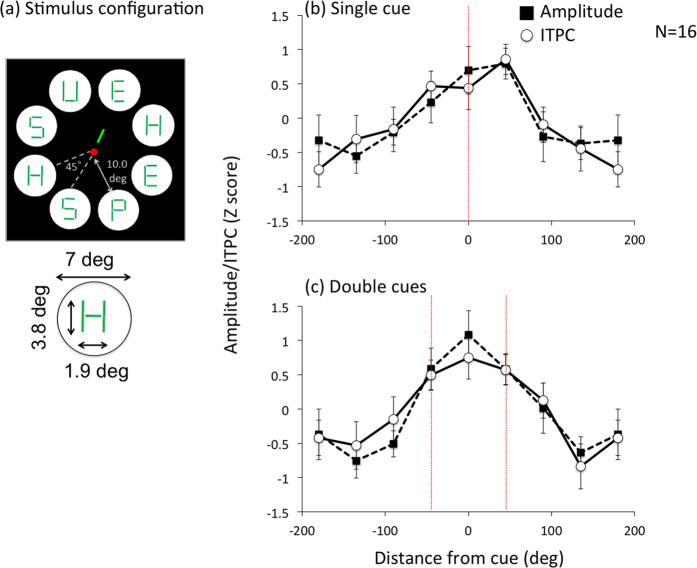
SSVEP amplitude and ITPC. (**a**) Stimulus configuration. Eight disks were arranged circularly around a central fixation point (red dot) and each one was 13.5° from the central fixation point. The luminance of each disk was modulated at a different temporal frequency. From the top right disk, which was pointed by a green bar in the figure, flicker frequency varied in the counterclockwise order 10.7, 11.4, 12.3, 13.3, 14.5, 16.0, 17.8, and 20.0 Hz in half of the sessions and 14.5, 16.0, 17.8, 20.0, 10.7, 11.4, 12.3, and 13.3 Hz in the other half. The RSVP task was detection of the target ‘H’ among distractors, U, E, and P at cued location(s). Letters were presented at all 8 locations and were replaced in synchrony every 187.5 ms. The letter presentation was in random order with a restriction of not presenting the target sequentially. (**b**) SSVEP amplitude and ITPC for each location relative to the cue for the Single cue condition. (**c**) SSVEP amplitude and ITPC results for the Double cue condition. The red dashed lines indicate the cue locations. The RSVP task was to identify the H at one cued location (indicated by a green bar) in Single cue condition or at either of two cued locations separated by a 90° rotation angle indicated by two green bars in the Double cue condition. Horizontal axis shows the distance from the cued location (**b**) or from the middle disk between the two cued disks (**c**).

SSVEP amplitude and ITPC were obtained from spectra (S1) in response to the flicker frequency of each disk and averaged over two occipital channels, O1, O2 (see S2 and S3 for other channels). To show attention modulation for each frequency, they were analyzed as a function of distance from the cue. Then, we averaged them after normalizing to Z score separately for each temporal frequency to eliminate the influence due to differences in sensitivity to different temporal frequencies. Both SSVEP amplitude and ITPC averaged over participants, again after normalizing to Z score for each participants, showed that attention spreads broadly over adjacent areas, further than 100° in rotation angle, or 25° in visual angle, when cued at one location (Fig. 1(b)). Attention also spread broadly over adjacent areas when cued at two locations 90 degrees apart (Fig. 1(c)) and peaked at a point midway between the two cued locations. A repeated three-way ANOVA test (Single/Double cues, amplitude/ITPC and location) showed that SSVEP response significantly varied with locations (F(7, 105) = 18.49, p < 0.001) and no significant difference was shown between the Single and Double cue conditions because their average was set to zero by normalization (no information of the test is give for such comparison hereafter). The pattern was almost identical for amplitude and ITPC (Fig. 1(b,c)). No significant interactions were found among location, cue condition, and SSVEP amplitude/ITPC.

**Figure 2:**
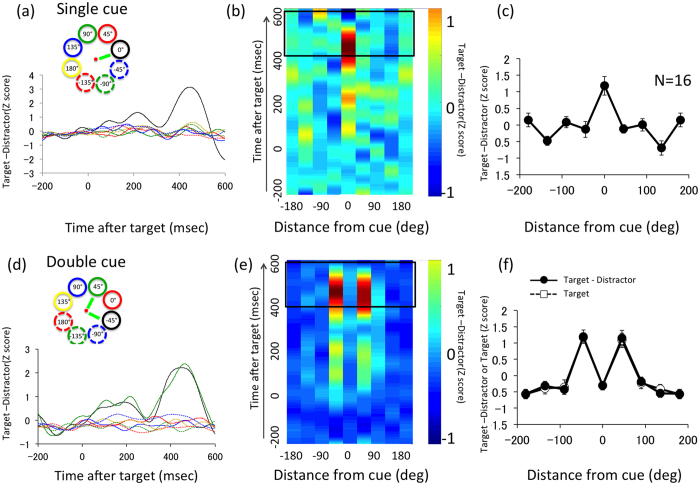
ERP and P3. (**a**–**c**) ERP results for the Single cue condition. (**a**) The difference between the potential evoked by the target presentation and that by the distractor at each of the eight locations, each represented by a different color (inset above). (**b**) The spatiotemporal representation of the evoked potential. Horizontal axis shows location relative to the cue and vertical axis shows time after the target presentation. (**c**) P3 component, defined as the average signal from 400 ms to 600 ms after the target presentation, for each location relative to the cue. (**d**–**f**) Same as in (**a**–**c**) but for the Double cue condition. White symbols in (**f**) indicate the same P3 obtained from ERP by target presentation.

We also analyzed ERP, extracting responses that were locked in time to the target presentation of RSVP sequences. The responses locked to distractor presentation were extracted for a comparison. The difference between the target and distractor ERP signals was calculated after averaging all target or distractor presentations of all trials (ERP to hit, that to target and that to miss are very similar to ERP to target-distractor while ERP to hit-miss did not show a large amplitude). Signals from the same two channels were used to compare with SSVEP (see S4 and S5 for other channels). Figure 2 show ERP results for the Single and Double cue conditions. The left panels (a and d) shows the ERP for each location and the middle panels (b and e) shows the spatiotemporal plot of the same data. We defined the P3 amplitude as the average signal between 400 and 600 ms, the epoch around which ERP signals are salient. The right panels (c and f) show P3 as a function of distance from the cued location. Contrary to SSVEP, the peak is sharp at the cued location for the Single (Fig. 2(a–c)) and there were two peaks corresponding to the two cued locations in the Double cue conditions (Fig. 2(d–f)). The results are not specific to channels used as shown in S4 and S5. A two-way ANOVA test (Single/Double cue conditions and location) revealed that the interaction between location and cue condition was highly significant (F(7, 105) = 4.72 p < 0.001) and that the main effect of cue condition was significant (F(7, 105) = 16.74, p < 0.001). The difference between the P3 amplitude at the cued location and the averaged amplitude of all uncued locations was significant by paired t-test (t(15) = 4.24, p < 0.001 for Single cue and t(15) = 6.00, p < 0.001 for Double cues).

It should be noted that there are SSVEP components for letter presentation cycle at 5.3 Hz (S1). Since the effect is similar for target and distractor ERPs, taking the difference between ERPs for target and distractor cancels out the components. To confirm that the 5.3 Hz components did not influence our analyses, we added the spatial tuning function of P3 of ERP for target presentation (white circle in Fig. 2(f)). The spatial tuning function for target P3 is virtually identical to the result of the difference between target and distractor.

The spatial profile of P3 was qualitatively different from that of SSVEP, as seen clearly in the Double cue condition. While P3 showed two peaks, one at each cued location, SSVEP exhibited only a single peak, suggesting that ERPs reflect selection of information by inhibiting or ignoring information at the non-target cued location. This is a strong piece of evidence for separate attentional processes with different spatial properties.

**Figure 3:**
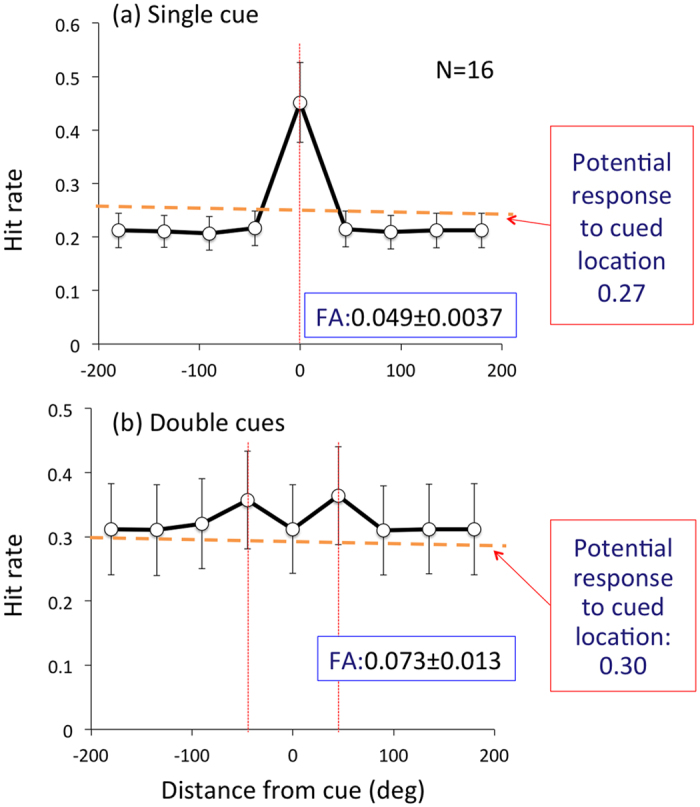
Behavioral results of RSVP. Hit rate for responses to target detection at each location relative to the cued location(s). Hit responses were calculated by counting the responses to the target (H) that was presented within a certain period of time after the target presentation. False Alarm rate is also calculated by counting the remaining responses. See Methods for details.

The behavioral results were consistent with the properties of P3 rather than SSVEP (Fig. 3). The detection rate for the RSVP target was high at only the cued location(s) and the rate at the other locations varied within 5% of the average. The detection rate at uncued locations was approximately the same as the erroneous detection rate estimated from the responses to the target at cued locations that happened to be presented at the same time as the target letter at another uncued location (see Method). A paired t-test showed a significant difference between the detection rate at cued locations and the rate averaged over all uncued locations (t(15) = 9.62, p < 0.001 for Single cue and t(15) = 6.86, p < 0.001 for Double cue conditions).

The neural generators of SSVEPs to luminance flicker have been reported in early visual areas including V1, V2, and V3^21^,^30^,^31^,^32^, while P3 reflects relatively higher level processing, where the visual system discriminate less frequently presented stimulus, (i.e., target) from more frequently presented stimulus, (i.e., distractors)^26^,^33^,^34^. These findings led us to speculate that SSVEP is related to signal enhancement, such as increase of gain or signal-to-noise ratio, at an early stage while P3 is related to a selection process for inhibiting or ignoring signals from distractors at a late stage. The similarity between the behavioral results and P3 responses is consistent with the hypothesis that P3 is related to a selection process. The RSVP tasks in Experiment 1 required selection of a target from distractors at adjacent regions.

If attentional spread at early stages has a broad spatial tuning, the behavioral results of a simpler task may show a broad spatial tuning, contrast to the narrow tuning of letter identification of RSVP task. We therefore repeated the experiment with an added simple secondary task, detection of luminance changes of the letter segments. One letter was darkened several times during a trial and the participants were asked to press a key when this luminance decrement was detected. The target detection in RSVP at cued locations was used as the primary task, using a key different from the one to indicate a luminance decrement. In addition, a larger spatial separation was applied to the original Double cue condition to investigate whether two peaks can eventually emerge in the SSVEP. This condition used two cues on opposite sides of the fixation point (termed the Double180° cue condition, as oppose to the original Double cue condition referred to as the Double90° condition here for clarity).

**Figure 4:**
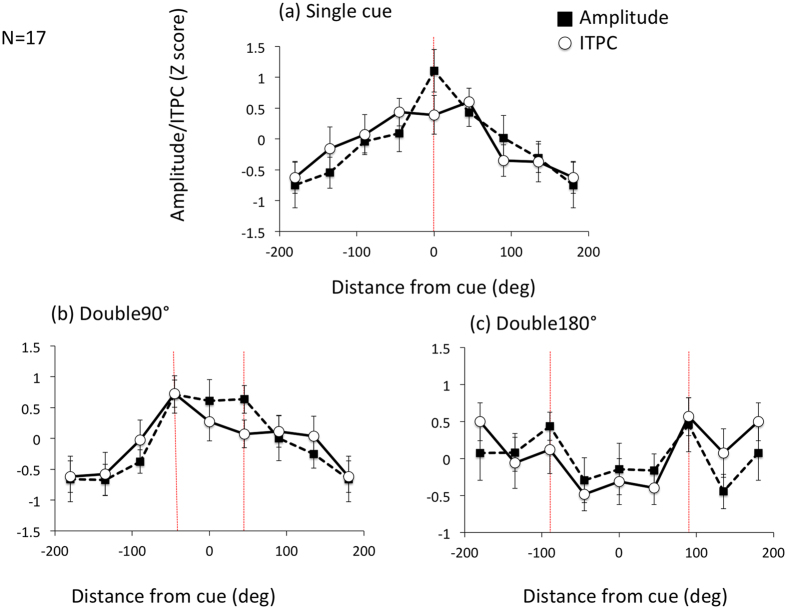
SSVEP amplitude and ITPC in dual task experiment. SSVEP amplitude and ITPC for the Single, Double90**°**, and Double180° cue conditions of Experiment 2. The notations are the same as in Fig. 1(b,c).

Broad spatial spread of attentional modulation was reproduced from SSVEP in both the Single and Double90° conditions, while there appeared to be two peaks at the two cued locations for the Double180° condition (Fig. 4). An ANOVA test showed a significant main effect of location (F(7, 112) = 9.56, p < 0.001) and significant interactions between location and cue condition (F(14, 224) = 5.90, p < 0.001). No significant interaction between location and SSVEP amplitude/ITPC. The significant interaction between location and cue condition indicates different shapes of spatial spread for the different cue conditions, which can be attributed to the shape of the Double180° condition different from the others’.

**Figure 5:**
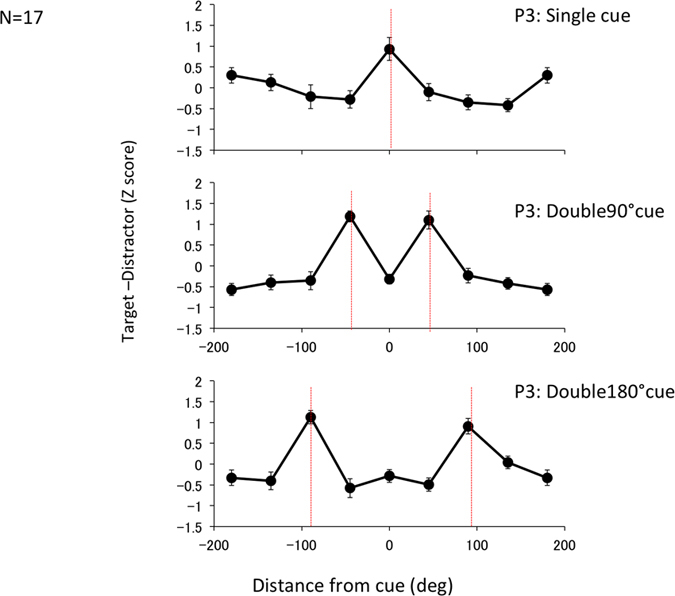
ERP and P3 in dual task experiment. P3 components of the ERP in Experiment 2. The notations are the same as in Fig. 2.

As in Experiment 1, P3 showed peaks at the cued location(s) for all cue conditions (Fig. 5). An ANOVA test showed a significant main effect for location (F(7, 105) = 2.84, p < 0.01) and interaction between cued location and cue condition (F(14, 210) = 8.48, p < 0.001). There was a significant difference between P3 at the cued location and P3 averaged over all uncued locations by paired t-test (t(17) = 10.56, p < 0.01 for Single, t(17) = 6.38 and p < 0.001 for Double90, and t(17) = 4.43 p < 0.001 for Double180).

**Figure 6:**
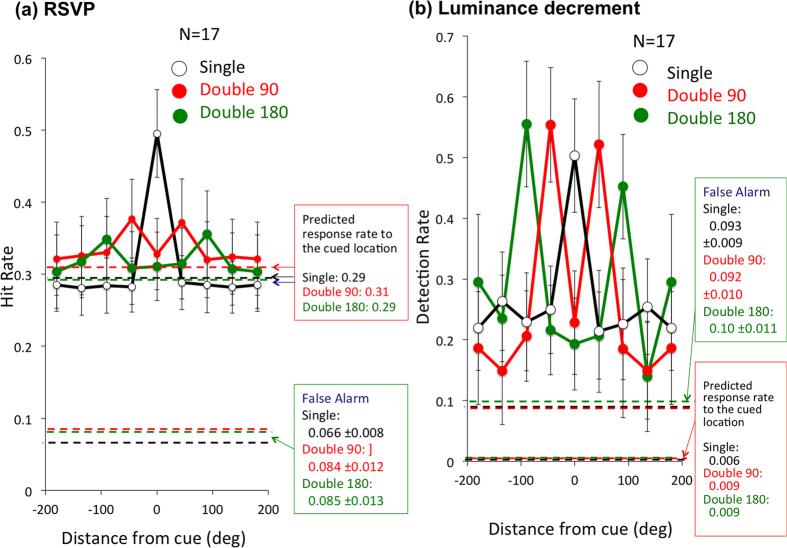
Behavioral results of RSVP and luminance change detection. (**a**) Hit rate for responses to RSVP target detection for each of the three cue conditions. (**b**) Hit responses for luminance change detection calculated by the same method used for the RSVP hit rate.

Behavioral results supported multiple stages of attentional modulation. The RSVP task responses were consistent with the P3 responses but distinct from SSVEP responses. Detection rate was high only at the cued location(s) for all cue conditions and approximately constant at other locations (Fig. 6(a)). The difference between the detection rate at the cued location(s) and the average over all uncued locations was highly significant by paired t-test (t(17) = 11.14, p < 0.001 for Single, t(17) = 9.41 p < 0.001 for Double90°, and t(17) = 6.33, p < 0.001 for Double180°). However, the results of detecting luminance change were different from those of RSVP target detection (Fig. 6(b)). First, although the luminance detection rate was prominent at the cued locations, the rates at other locations were clearly above the chance and the erroneous detection rate calculated as for the RSVP task. Attentional resource should have been distributed broadly over uncued locations to detect the luminance change there. Second, the luminance detection rate at cued locations was similar for different cue conditions (all about 0.5), in contrast to the change in detection rate among the cue conditions in the RSVP, which was highest for Single, second highest for Double90°, and the lowest for Double180° conditions. Attending to two locations had little influence on the rate of luminance change detection, but impaired RSVP task performance, and the effect was worse when the separation of cued locations was larger. Thus, RSVP detection is more strongly influenced by cue conditions than is detection of luminance changes, strongly suggesting that different attentional processes are responsible for performance of the two tasks.

The assumption of attentional modulation at multiple stages is consistent with previous results suggesting separate attentional processes mediated by different cortical areas. Early processes tend to respond to visual stimulation and later processes tend to show more influence of top-down control^35^. A previous report demonstrated attention modulation with broad spatial tuning in early visual cortices^36^. Selection of attended location and/or inhibition of adjacent areas have also been shown at relatively later stages controlled by higher visual centers such as the frontal eye field^37^ and lateral intraparietal cortex^38^,^39^,^40^. These results are consistent with a multistage attention mechanism. The significant contribution of the present finding to the idea of the multistage attention mechanism model is that different stages have different spatial properties, which is likely related to different attention functions.

An alternative to multiple processes is a unitary attentional process in which spatial attention depends on task load. For example, an easy task could allow attention to spread broadly while a difficult task could force the focus of attention toward the center, resulting in different effects of attention on task performanc^41^. However, our experiments revealed different spatial tuning for behavioral and physiological results in the same participant and within the same experiment. Thus, the present results cannot be explained by different attentional states due to task difficulty.

## Discussion

### Period of data for analyses

We used EEG signals from different periods of time for SSVEP and ERP because SSVEP and ERP reflect attentional effects differently. That is, where in the EEG signal the attentional effect is found is different. On one hand, effect of attention should be found in SSVEP when the participant is attending on a location, that is, any time during a trial in the present experiment. On the other hand, attentional modulation on ERP should be found after target presentations, by definition, when the target is presented any time during a trial. Both measures were averaged over the entire period of a trial. SSVEP was obtained using EEG signals throughout each trial and ERP was an averaged of data after all target presentations, independently on the time of presentation. In both SSVEP and ERP, response is expected to be high at the attentional focus throughout a trial because the participants kept paying attention on the cued location(s) to do RSVP task. Theoretically, therefore, it is reasonable to use EEG signals from different periods of time for SSVEP and ERP analyses. It is, however, an important and interesting question how target presentations might influence SSVEP. An SSVEP analysis of EEG data measured after target presentation provided the answer (see Shift of attention).

### Lateral inhibition

Narrow spatial tuning can be realized by inhibition at adjacent locations (lateral inhibition) or ignoring information at locations other than cued locations (selection at attention focus^42^). To examine whether there is lateral inhibition, we compared the P3 amplitude close to and more distant from the cue (close: 45° apart from a cue; more distant: 90° apart). For example, we compared the average of ±90° and 0° (45° from the cue(s)) and that of ±135° (90° apart from the cue) in 90° Double cue conditions). No condition of either experiment showed statistically significant difference between 45° and 90° locations although the average values were larger in 90° from the cue in some conditions. We also examined the P3 amplitude in response to distractor presentation to detect possible inhibitory effects on distractors (S6). Although amplitude variation among different locations was very small, clear reductions were observed at cued locations (a t-test showed significant difference between cued and uncued locations: p < 0.05 for all conditions). These analyses suggest that a selection process at the cued location is the major factor narrowing the attentional extent but that inhibition of distractors at the cued location also plays a role in narrowing the attentional extent.

### Shift of attention

**Figure 7:**
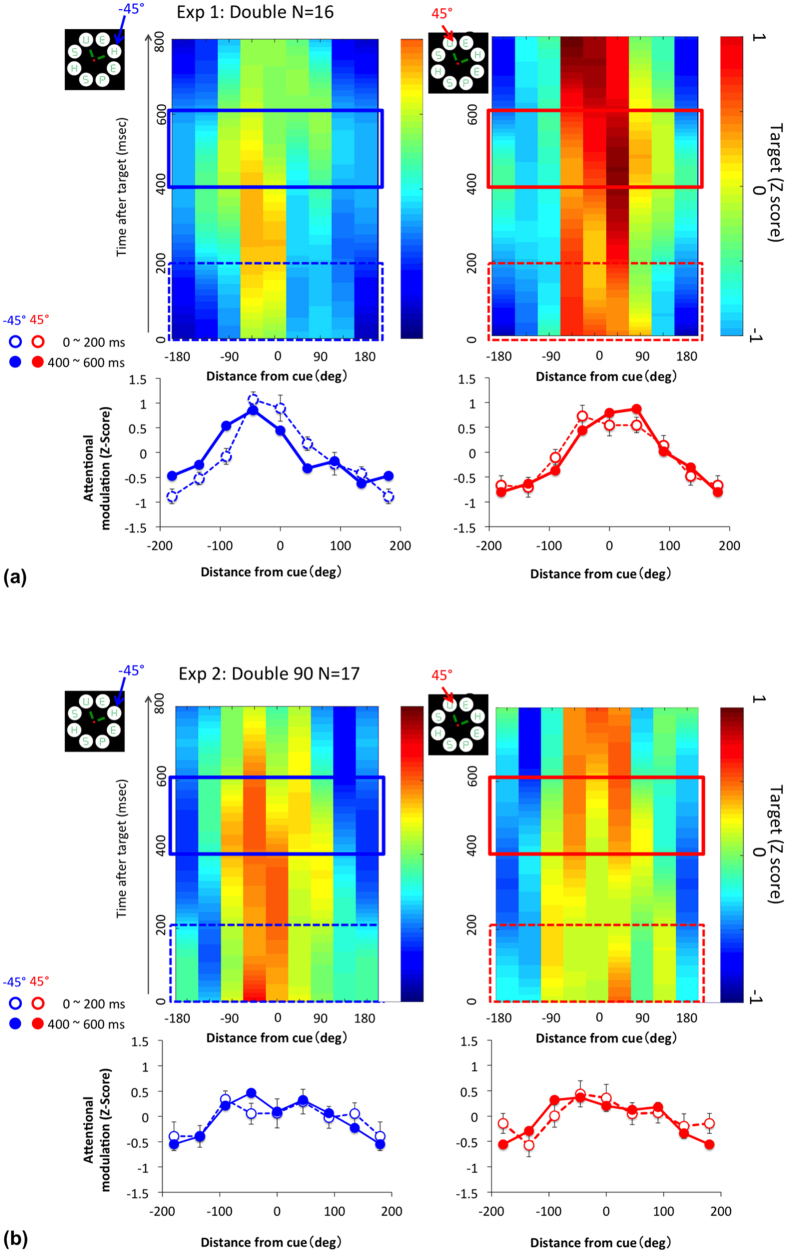
Event Related SSVEP. SSVEP amplitude locked to the target presentations at the two cued locations of the Double of Experiments 1 (**a**) and Double90° of Experiment 2 (**b**). The spatiotemporal responses for SSVEP amplitude (top panels). Average amplitude for right after target presentation (0–200 ms) and for P3 period (between 400 and 600 ms after the target). Different colors indicate the different target locations. Left panels are for −45° and right panels are for 45°.

To confirm stability of attention on cued location without re-direction to other disks and of spatial property after target presentation, we analyzed temporal change of SSVEP after target presentation (i.e., the “Event Related SSVEP” (ERSSVEP)). Figure 7 shows the ERSSVEP amplitude right after (0~200 ms) and 400 ms after (P3 period) target presentation in the Double cue condition of Exp. 1 (Fig. 7(a)) and Double90° cue condition of Exp. 2 (Fig. 7(b)). The data represent SSVEP amplitude at each location and time after target presentation at a cued location. Left panels show the response after target presentation of −45° and right panels show that of 45°. If target attracts attention, the peak should shift to −45° (left) or 45° (right) and the shape of spatial tuning should become different between the initial and P3 periods. An ANOVA showed that significant interaction between period (initial vs P3) and location for Experiments 1 (F(7, 105) = 2.83. p < 0.05) but not for Experiment 2 (F(7112) = 1.05. p > 0.5), whereas the main effect of location was significant ((F(7,105) = 12.38. p < 0.001 for Experiment 1 and F(7, 112) = 5.65. p < 0.001 for Experiment 2). These results may suggest shift of attention to the target location. However, the spatial property can be characterized as a broad tuning even with the possible shift of attention to the target location. That is, attentional spread shown by SSVEP is relatively stable throughout a given trial including around the time of target presentation while there may be some effect of target on SSVEP. Although extremely quick shifts of attention cannot be ruled out based on the present results, empirical studies have shown that it takes 200 ms or more to shift attention endogenously^18^,^19^,^34^,^41^.

### Division of attention

The clear separation of the P3 peaks and hit rates in the Double cued RSVP task suggests division of attention. The double peak suggests that attention focuses on two locations. The present experiments were not designed to examine whether spatial attention was divided, so the double peaks could be interpreted as stemming from trial-by-trial variation of attention focus. However, the small effect of target presentation (Fig. 7) indicates that attention was stable over each trial, covered two locations simultaneously, so the double peaks can be attributed to attentional division.

The double peaks, and attentional division also, may be partially due to hemisphere effect, which is the advantage of visual processing of two stimuli presented in the left and right visual field over those presented in the same visual field. The hemisphere effect can be explained by assuming separate attention resources for the left and right hemispheres^43^,^44^,^45^,^46^. Performance on tasks involving visual stimuli presented across left and right visual fields is often better than that on the same task using the same stimuli but presented in only one visual field. Our experiment, however, showed little hemisphere advantage. The comparison of attentional spreads of SSVEP between the within and across hemi-fields conditions (S7) showed similarly broad spatial spread with a peak midway between the two cues in general, while there may be two small peaks and broader spatial spread for across SSVEP in the Double90° condition of Experiment 2. Two-way ANOVA showed no significance for the difference between across and within SSVEPs in Experiments 1 and 2, that is, no interaction between the visual field condition and location (F(7, 105) = 0.301, p = 0.95 in Experiment 1 and F(7, 112) = 1.65, p = 0.13 in Experiment 2). ERP showed similar results for across and within conditions (S8). P3 amplitude and the detection rates at cued locations were not significantly different between across and within conditions. P3 amplitude (Z-score) at cued location for the across and within conditions were 0.96 and 1.02 (t = 0.73, p = 0.49) in Exp. 1 and, 0.86 and 0.93 (t = 0.53, p = 0.69) in Exp. 2. The detection rates showed significant difference between across and within conditions, while the difference is small. Detection rate was 0.37 and 0.35 (t = 5.63, p < 0.001) in Experiment 1 and 0.38 and 0.37 (t = 2.45 p < 0.05) in Experiment 2. These results indicate that the hemisphere advantage was smaller than the effect of cue location in our experimental condition. Since behavioral results showed only small hemisphere advantage, it is not surprising not to obtain a hemisphere effect in EEG signals.

### Spatial attention at multiple stages

The present results showed different spatial properties between SSVEP and P3, suggesting that there are two stages with different attentional spatial tunings in the visual system. Is the difference of task relevance between disks and letters the cause of the difference in spatial property? Flickering disks had no relationship with the RSVP task and attention may not be necessary on the disks. The difference may have caused the difference in spatial property between SSVEP and P3. However, SSVEP results showed attentional modulation on disks even they were irrelevant to the task. The difference between the two measures is likely related to two different attentional processes with different spatial properties. Although, strictly speaking, SSVEP may not be the process of spatial attention, assuming contribution of spatial attention that modulates SSVEP signals is one of the simplest interpretations of the present results.

**Figure 8:**
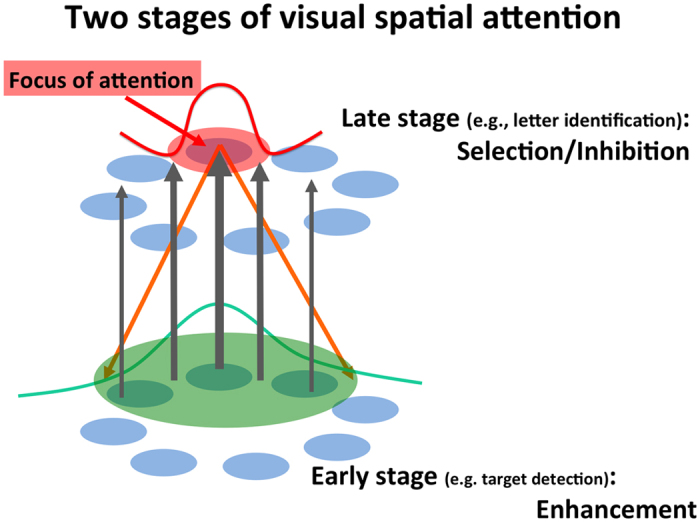
Two-stage model of spatial attention. A two-stage model of spatial attention. Blue disks are representations of visual stimuli at early and late stages of visual processing. A top-down process marks an area to attend for spatial selection at the late stage (red circle). Attention effect spreads over to adjacent areas at the early stage through feedback connections (orange lines), resulting in a large spatial extent (green circle). The attentional modulation provides enhancement (green line) of output signals to feed the later stage (gray arrows). The signal is filtered at the later stage (red line) to select information at the attention focus.

Multistage models of attention have been proposed^14^,^15^,^16^. Franconeri *et al*. proposed a framework that assumes similar spatial tuning in a variety of feature dimensions in the visual system and Hopf *et al*. described multiple stages with similar but scaled attentional filters. Since they assumed an attention filter with the same shape for all stages, they cannot explain our results. We propose a two-stage model with different attentional spatial tunings (Fig. 8), to interpret the distinct effects on SSVEP and P3 and between RSVP target detection and luminance change detection. In Fig. 8, blue disks are a representation of visual stimuli at an early stage and a late stage of visual processing. A top down process marks a location to attend in a space representation at the late stage (red circle). The attention effect spreads over to adjacent areas at the early stage through feedback connection (orange lines), resulting in a large spatial extent (green circle). Attentional modulation provides enhancement (green line) of output signals to feed the late stage (gray arrows). The signal is filtered at the late stage (red line) to select information at the attention focus. Although this is a conceptual model to describe the two stages of attention found in the present study, the multistage model can explain both the attention spread and the selection at the attention focus.

Parietal cortex is often assumed to be the area to control visual attention and the attention signals are transmitted down to visual cortexes^47^,^48^,^49^,^50^,^51^, with which our model is consistent. In addition, there are studies that showed broad spatial tunings of attention enhancement in early visual brains. The full width at half maximum (FWHM) of about 60° in rotation angle for V1 and larger in higher visual areas (about 100° for LO)^36^,^52^,^53^. These sizes of spatial spread are about the same as ours (see Fig. 1(b,c)) and are consistent with the broad spread of spatial attention. Moreover, attentional selection is known between two stimuli within the receptive field (RF) in V4 neurons^54^,^55^,^56^ A target letter can be selected from distractors in a RF by attentional modulation for certain features. The simple form of the selection process of V4 neurons, however, cannot explain our results because the selection is based on visual features, not location. The selection process in our model may be related to an interaction between feature based selection and top-down signals for spatial selection from higher areas on V4 neurons^56^,^57^,^58^.

## Method

We conducted two experiments with the same stimuli and procedure except for two differences. First, Experiment 1 used an RSVP task while Experiment 2 added an additional detection task to the RSVP task. For RSVP, the participant pressed a key when he detected a target letter, H, at a cued location during a RSVP sequence. For the detection task in Exp. 2, one letter was darkened several times during a trial and the participants were asked to press another key for detection. Second, there are two attention conditions in Exp. 1 (cued at a single location and cued at two locations 90° apart) while three attention conditions in Exp. 2 (the same two conditions as in Exp. 1 and additional condition with cues at two locations 180° apart).

### Participants

Eighteen with normal or corrected-to-normal visual acuity gave informed consent to participate in Experiment 1 (age 24.4 ± 3.7, all male) and in Experiment 2 (age 24.0 ± 3.4, all male). Thirteen of them participated both Experiments 1 and 2. Data from two participants from Experiment 1 and those from one were excluded due to excessive artifacts in the electro-oculogram (EOG) data (see Analysis). The experiments were approved by the Ethics Committee of Research Institute of Electrical Communication, Tohoku University, and the methods were carried out in accordance with the approved guidelines. All participants gave written informed consent.

### Stimuli

The stimulus configuration is shown in Fig. 1. Eight disks arranged circularly around a central fixation point flickered at different temporal frequencies: 10.7, 11.4, 12.3, 13.3, 14.5, 16.0, 17.8, and 20.0 Hz. These frequencies were selected to be clearly visible with 50% luminance contrast and not to have the same harmonics. The average luminance of the disks was 76.3 cd/m^2^. Letters were presented within each disk in green (Exp. 1) or red (Exp. 2). Experiment 2 used red letters for better visibility to compensate for the increase of task difficulty due to an additional secondary task. The luminance and color coordinates of the green were 104.4 cd/m^2^ and (0.284, 0.597), and those of the red were 32.4 cd/m^2^ and (0.620, 0.336). The scale of the letter was 1.9° in width and 3.8° in height, and the disk diameter was 7.0°. The distance of each disk from the fixation point was 13.5° from center to center. We used four letters, all of which were made of 5 line segments: H as the target and U, E and P as distractors.

### Procedure

Letters changed among four (H, U, E, and P) every 187.5 ms and the task was to detect the target letter H, which was presented 7 times on average during a 6-s trial. Green bar(s) were presented before the trial as cue(s) to indicate the disk(s) to attend. A key press by the participant started the trial when he was ready to do the task at the cued location(s). Then, disks started flickering and letters started changing. The participant pressed a key when he detected a target H at the cued disk (Single cue condition) or at either of the two cued disks (Double cue condition) during the trial. Before and after the trial, disks were stationary and all contained the number 8 (all seven segments were colored). The cued location(s) were changed from trial to trial so that all disks were cued the same number of times during a session. Six (Exp. 1) or four (Exp. 2) sessions of 56 trials were performed for each condition and by each participant. There were two different flicker conditions, each used for half of the sessions. In one flicker condition, the flicker frequency of the top right disk was set to 10.7 Hz, and the other flicker frequencies in the counterclockwise direction were 11.4, 12.3, 13.3, 14.5, 16.0, 17.8, and 20.0 Hz. In the alternate flicker condition, the counterclockwise order from top right was 14.5, 16.0, 17.8, 20.0, 10.7, 11.4, 12.3, and 13.3 Hz.

### EEG Recording

Following the procedure used in Kashiwase *et al*.^18^, we recorded brain electrical activity from 19 scalp electrodes mounted on an elastic cap connected to an EEG recording system (Neurofax EEG-9100, Nihon Koden, Tokyo, Japan). The electrode arrangement was based on the International 10–20 System with reference electrodes placed on both ear lobes. EEG signals were recorded with a 120 Hz high-pass filter, digitized at 1000 Hz, and stored for off-line analyses. All electrode impedances were confirmed to be below 5 kΩ before each session. To ensure that any SSVEP modulation observed in the experiment was attributable to an attentional effect, participants were asked to fixate on the center of the display and try not to blink during each 6-s trial. We also recorded horizontal and vertical eye movements based on signals from two additional electrodes placed 1 cm above and below the outer canthus of the left and right eyes. We excluded trials with EOG deflections of more than ±50 μV, which corresponds roughly to a 5° eye shift. This also removed trials with eye blinks. Data from 2 of 18 participants in Experiment 1 and 1 of 18 in Experiment 2 were removed from the analysis because of high rates of trial rejection. The number of rejected trials for the rest of the participants was less than 10% in Experiment 1 and less than 20% in Experiment 2 and the average rejection rate of these participants was less than 5% in both experiments.

### Hit rate

We evaluated the target detection rate of the RSVP task as follows. We first computed the histogram of the response time (RT) to targets (S9), counting the time from target (‘H’) presentation closest to each key press. Then, we determined the time window for the response to be judged a “hit” by fitting a Gaussian function to the RT distribution. Using this function, responses in the time window ± 2σ from each target onset were regarded as “hits” and all others as “false alarms (FAs)”. We calculated the FA rate as the percentage of FAs against all distractors presented during a trial, which is the number of responses outside the hit temporal window (±2σ) over the total number of letter presentations (30 per trial) minus the number of target presentations (7 per trial on average). To evaluate the attentional modulation of behavioral performance, we plotted the Hit rate at each disk location relative to the cued location (Figs 3 and 6) for each condition. The RT distribution was obtained separately for different cue conditions for each participant and used for calculation of hit rates. The average and standard deviation of duration for hit responses was 593 ± 39.6 ms in Experiment 1 and 561.1 ± 15.9 ms in Experiment 2.

The target letter was sometimes presented at two locations simultaneously, so a certain number of responses related to the target at uncued locations were actually responses to the target at the cued location, which should not be regarded as target detection at an uncued location. The probability, *p*_*c*_, which we consider as the chance level for uncued locations, is obtained by


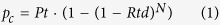


where *Pt* is the detection rate of the target at cued location(s), *Rtd* is the target presentation rate at each uncued location with a simultaneous target presentation at the cued location, and *N* is the average number of target presentations during the RT range, within which a key response is regarded as a response to the target presentation at the cued location. *Pt* values were 0.45 and 0.37 for the Single and Double cue conditions in Experiment 1, *Rtd* was defined as 0.25 in the experiment (random presentations of four letters), and the *N* values were 3.17 for both cases, calculated by dividing the hit RT range (592.9 and 592.5 ms) by target presentation duration (187.5 ms). The hit RT range for the Single and Double cue conditions is a ±2σ of a Gaussian distribution function that approximates the RT distribution for each condition. Estimated *p*_*c*_ values were 0.27 and 0.31, respectively, for the Single and Double cue conditions. In Experiment 2, *Pt* values were 0.50, 0.37, and 0.35 for the Single, Double90°, and Double180° cue conditions, respectively, and corresponding *N* values were 3.05, 2.98, and 2.97, calculated as above from the individual task hit RT ranges (570.6, 557.2, and 555.5 ms). Again, the hit RT range was ±2σ of a Gaussian distribution function that approximates the RT distribution of each condition. Estimated *p*_*c*_, values were 0.29, 0.30, and 0.29 for the Single, Double90°, and Double180° cue conditions.

### Data Analysis

To analyze the frequency characteristics, EEG data for each trial were transformed to the frequency domain by fast Fourier transform using a 20% tapered cosine window, which yielded a frequency resolution of 0.5 Hz.

The Fourier component F for a temporal frequency *f* is given by


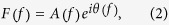


where *A* and *θ* are amplitude and phase. Amplitude is computed as the average of the absolute Fourier coefficients obtained from individual trials. As an index of phase coherence, intertrial phase coherence (ITPC) was calculated.


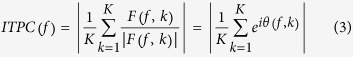


ITPC represents the degree of phase alignment of each EEG component across trials, which is a real value between 0 (uniform phase distribution across trials) and 1 (perfect phase synchronization across trials).

Data were normalized separately for each temporal frequency to eliminate the influence due to differences in sensitivity to different temporal frequencies. The amplitude changes as a function of the distance between the corresponding disk and the cued location, and the change can be attributed to a pure attentional effect because the physical conditions are identical among the different cue locations. After calculating a Z-score to normalize the data for each frequency, we averaged amplitude/ITPC score of all temporal frequencies, in order to minimize the effect of different signal strengths among temporal frequencies. We then calculated Z-score again for each participant from averaged data over different frequencies, to normalize the individual differences of attentional modulation.

We focused on the channels of two occipital electrodes (O1, O2) based on the finding that these channels showed the large attentional modulation (S6). The data from the channels were averaged for the analysis. The analysis with seven electrodes with large modulation seen in S6 (O1, O2, T5, T6, P4, P3 and Pz), following a previous SSVEP study of our laboratory^18^, showed basically the same results of both SSVEP and ERP (not shown). We also analyzed the data with amplitude ratio between a peak frequency and adjacent frequencies and found no systematic differences from the analysis shown (not shown). The similar results are expected because data are normalized for each frequency to avoid the effect of sensitivity differences to different temporal frequencies.

To estimate spatial spread of attention from the ERP to the RSVP target, we analyzed the data as follows. First, we averaged the ERP data to targets presented and removed temporal frequencies higher than 10 Hz by low-pass filtering to eliminate the influence of SSVEP responses. Then, we calculated the difference between the ERPs to target and those to distractors. In the averaged waveform of target − distractor, where the baseline was defined as the mean amplitude between 150 ms before and the time of target presentation, we found largest attentional modulation at around 500 ms for both Single and Double cue conditions in Experiment 1 (Fig. 4). Based on this observation, we defined the magnitude of the P3 component as the average between 400 and 600 ms after target presentation (solid rectangle in Fig. 4) for each participant. We calculated Z-scores for each participant and averaged them to obtain spatial property of attentional modulation in Fig. 4(c,f).

The average number of events used to obtain ERP function for each participant was 1254 Experiment 1 and 840 in Experiment 2.

## Additional Information

**How to cite this article**: Shioiri, S. *et al*. Visual attention spreads broadly but selects information locally. *Sci. Rep.* **6**, 35513; doi: 10.1038/srep35513 (2016).

## Supplementary Material

Supplementary Information
