# Corrigendum: Visual attention spreads broadly but selects information locally

**DOI:** 10.1038/srep46198

**Published:** 2017-04-07

**Authors:** Satoshi Shioiri, Hajime Honjyo, Yoshiyuki Kashiwase, Kazumichi Matsumiya, Ichiro Kuriki

Scientific Reports 6: Article number: 3551310.1038/srep35513; published online: 10 19 2016; updated: 04 07 2017

The original version of this Article contained errors in the Supplementary Figures 2, 3, 4 and 5, where the EEG data analysed was relative to the average of the C3 and C4 electrodes. These errors do not affect the conclusions of the Article and have now been corrected in the Supplementary Information file that accompanies this Article.

