# Supplementary material for: Visual attention spreads broadly but selects information locally
Source: Sci Rep. 2016 Oct 19;6:35513. doi: 10.1038/srep35513 (PMC5069499; doi:10.1038/srep35513)
Supplement: Supplementary Information [file srep35513-s1.pdf]

Visual attention spreads broadly but selects information locally

Satoshi Shioiri, Hajime Honjyo, Yoshiyuki Kashiwase, Kazumichi Matsumiya, Ichiro Kuriki
Research Institute of Electrical Communication, Tohoku University

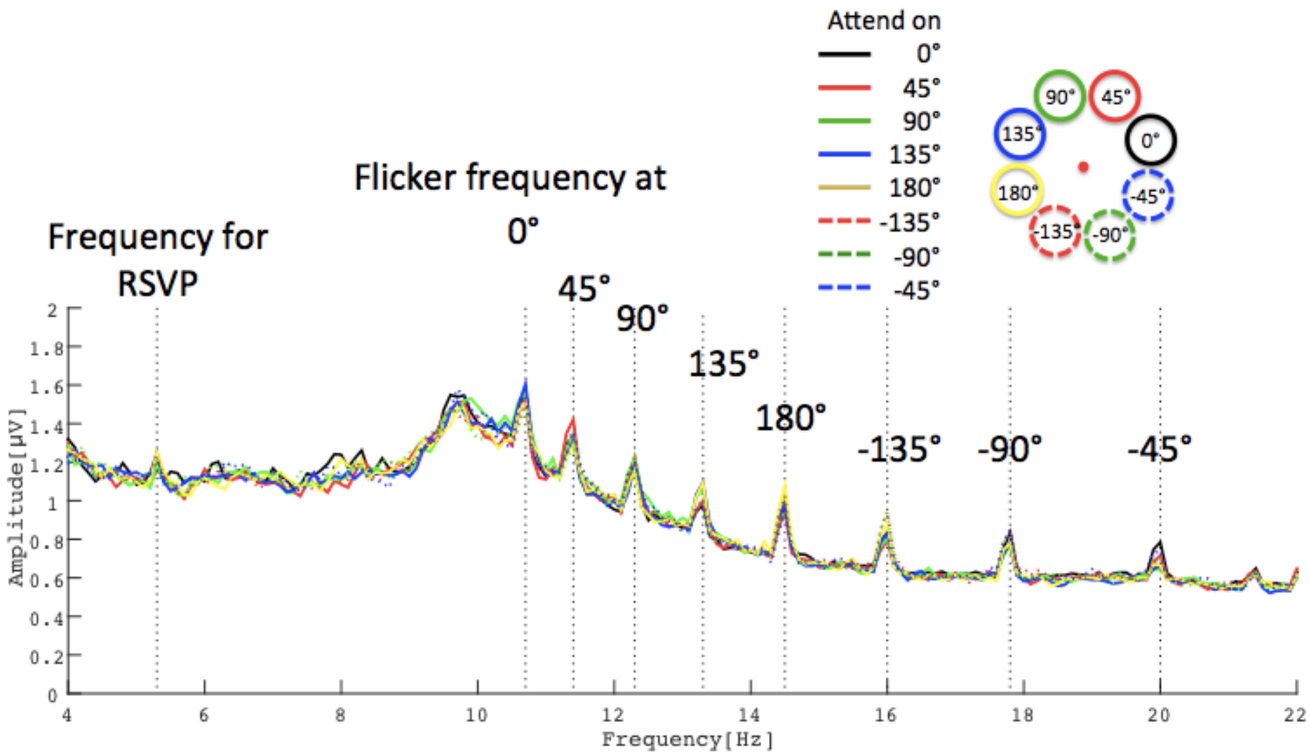

Supplementary Figure 1

Spectra for the Single cue condition of Exp. 1 obtained from average results of three sessions with the frequency arrangement as shown (the top right frequency was 10.7 Hz, and increased in the counterclockwise direction). Different colors indicate different cue conditions. The amplitude of each frequency tends to be larger when the location corresponding to the frequency is attended (e.g., red at 45° and green at 90°). Amplitudes of each frequency were averaged over four channels of all participants. Clear peaks are shown at SSVEP frequencies. The difference between the peak value and the adjacent values (±0.1 Hz) is statistically significant when tested with a t-test ($p < 0.01$ for all frequencies)

Exp1: Single N=16
SSVEP Amplitude

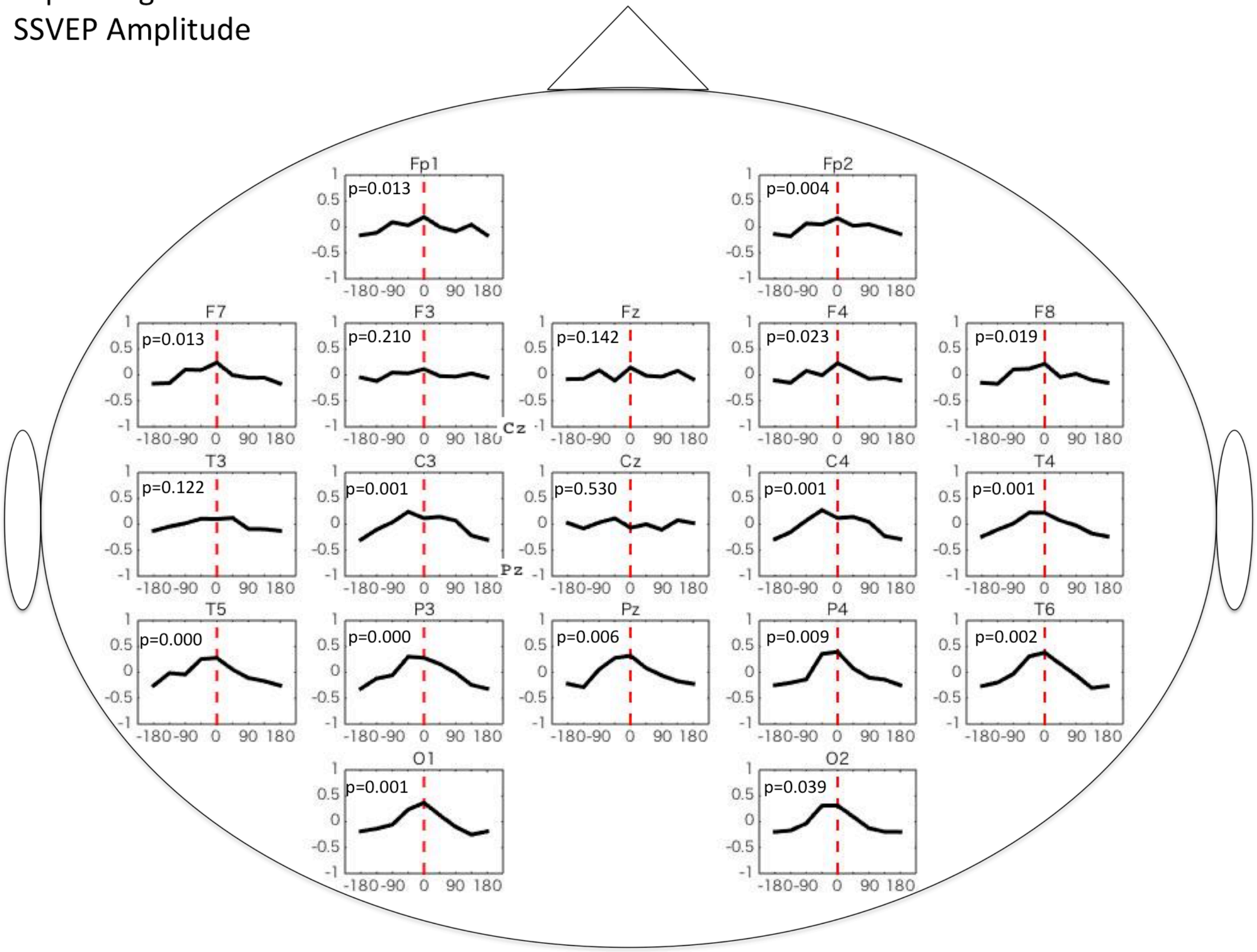

Supplementary Figure 2

SSVEP amplitudes for each channel of the Single cue condition of Experiment 1 averaged over participants. P value at each panel represents the significant level of t test for the difference between cued (0°) and ignored (180°) locations.

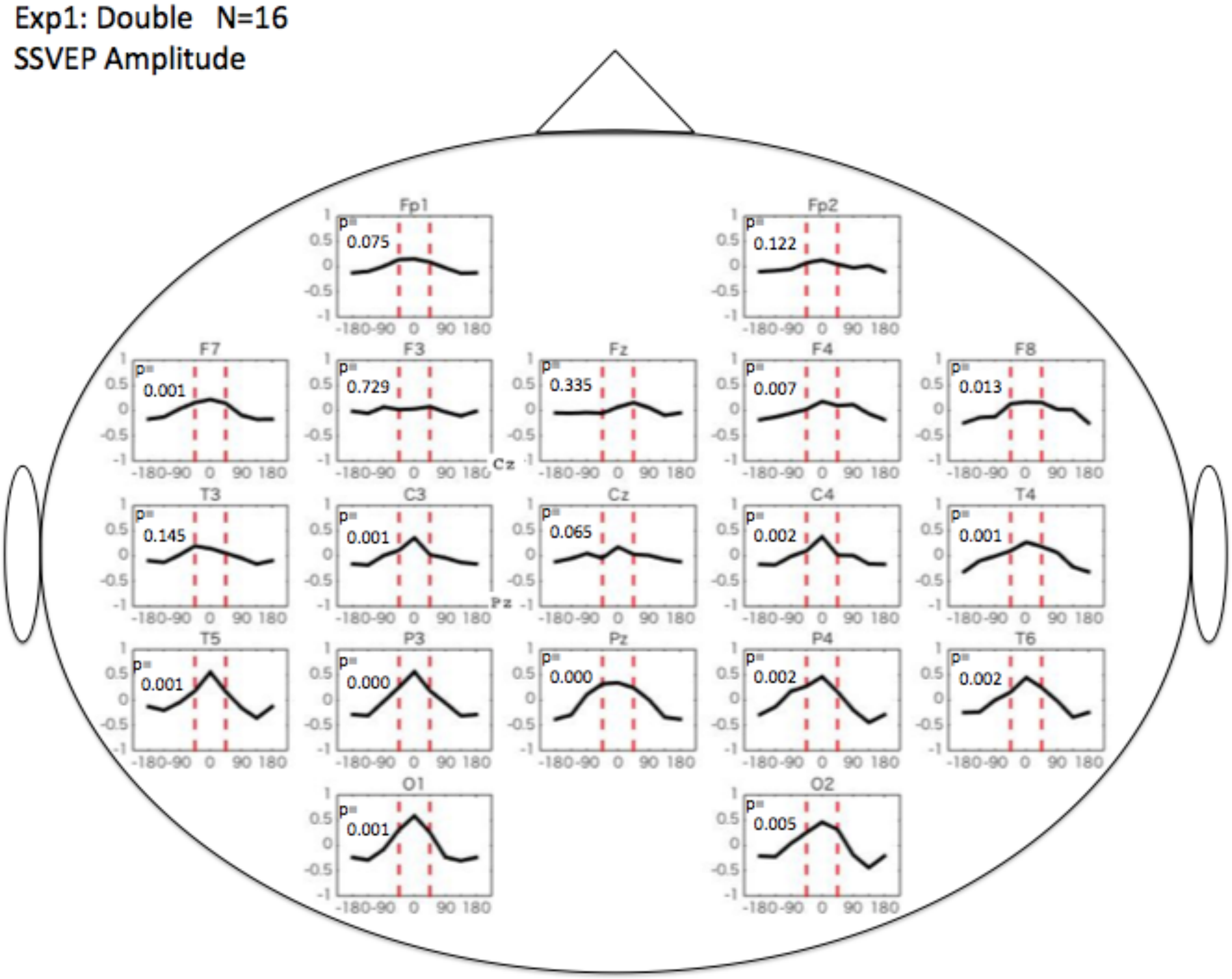

Supplementary Figure 3

SSVEP amplitudes for each channel of the Double cue condition of Experiment 1 averaged over participants. P value at each panel represents the significant level of t test for the difference between the midpoint of the two cued locations (0°) and ignored (180°) location.

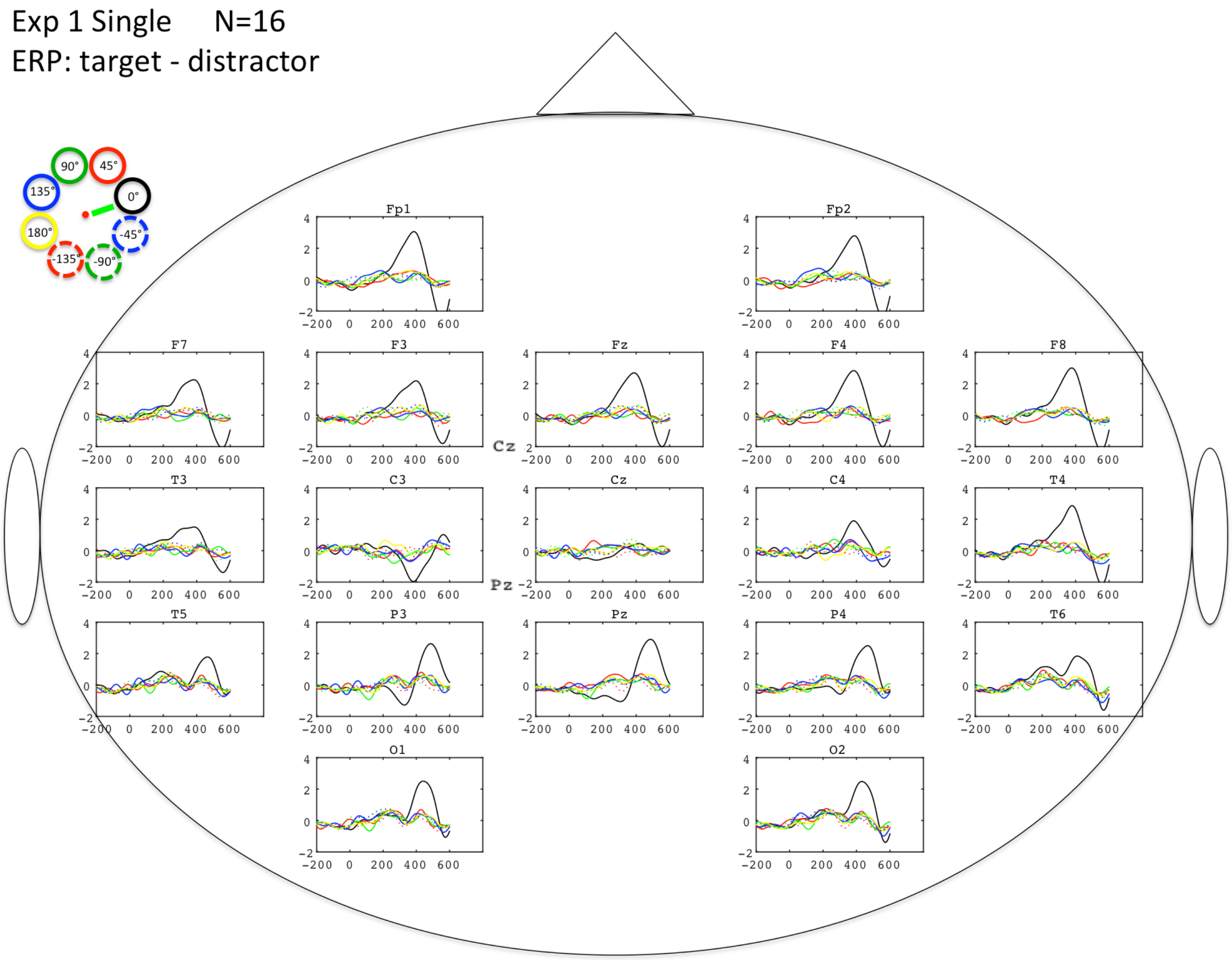

Supplementary Figure 4

ERP function for each channel of the Single cue condition of Experiment 1 averaged over participants. Different colors indicate different target presentation location relative to cue.

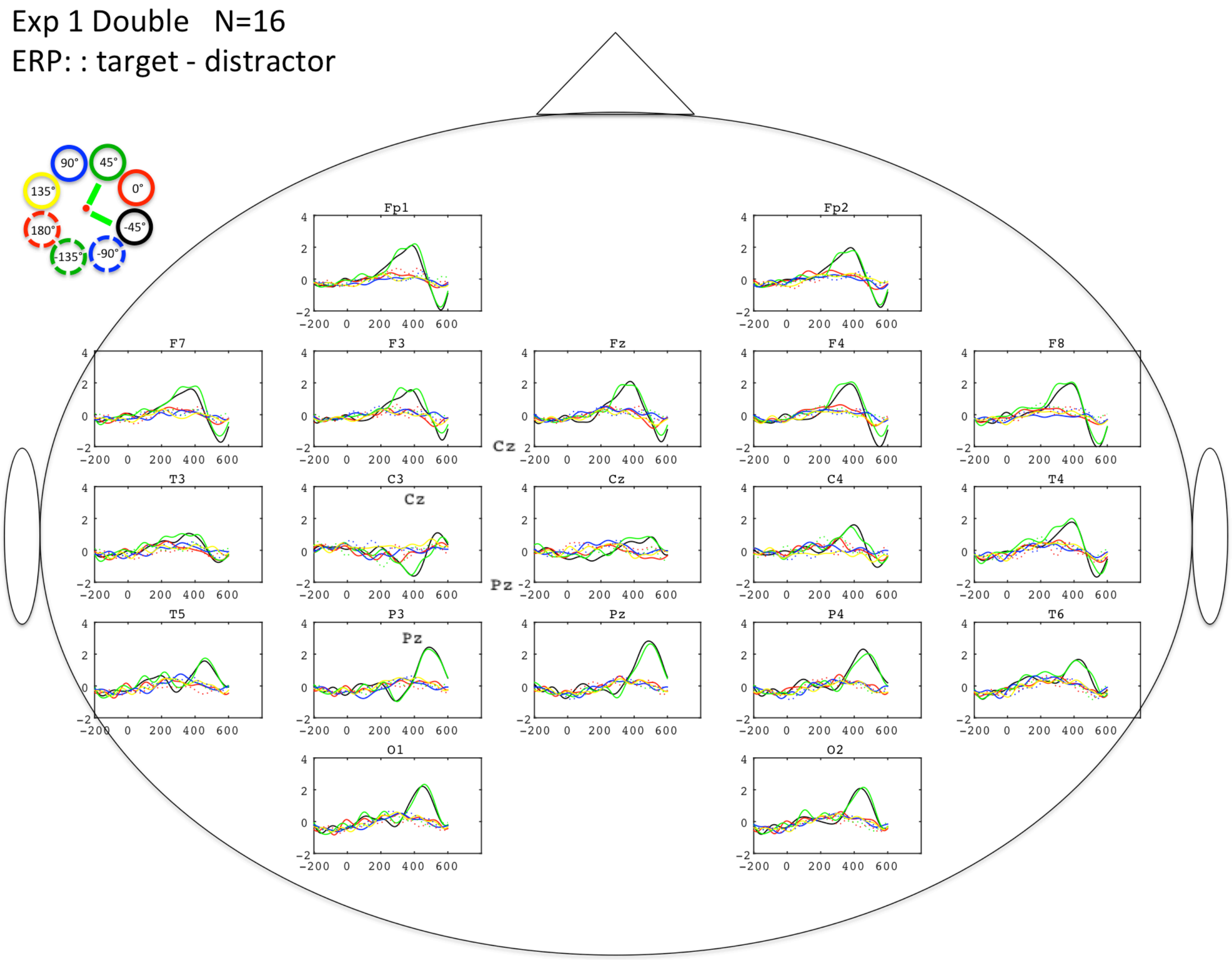

Supplementary Figure 5
The same ERP function for each channel but for the Double cue condition.

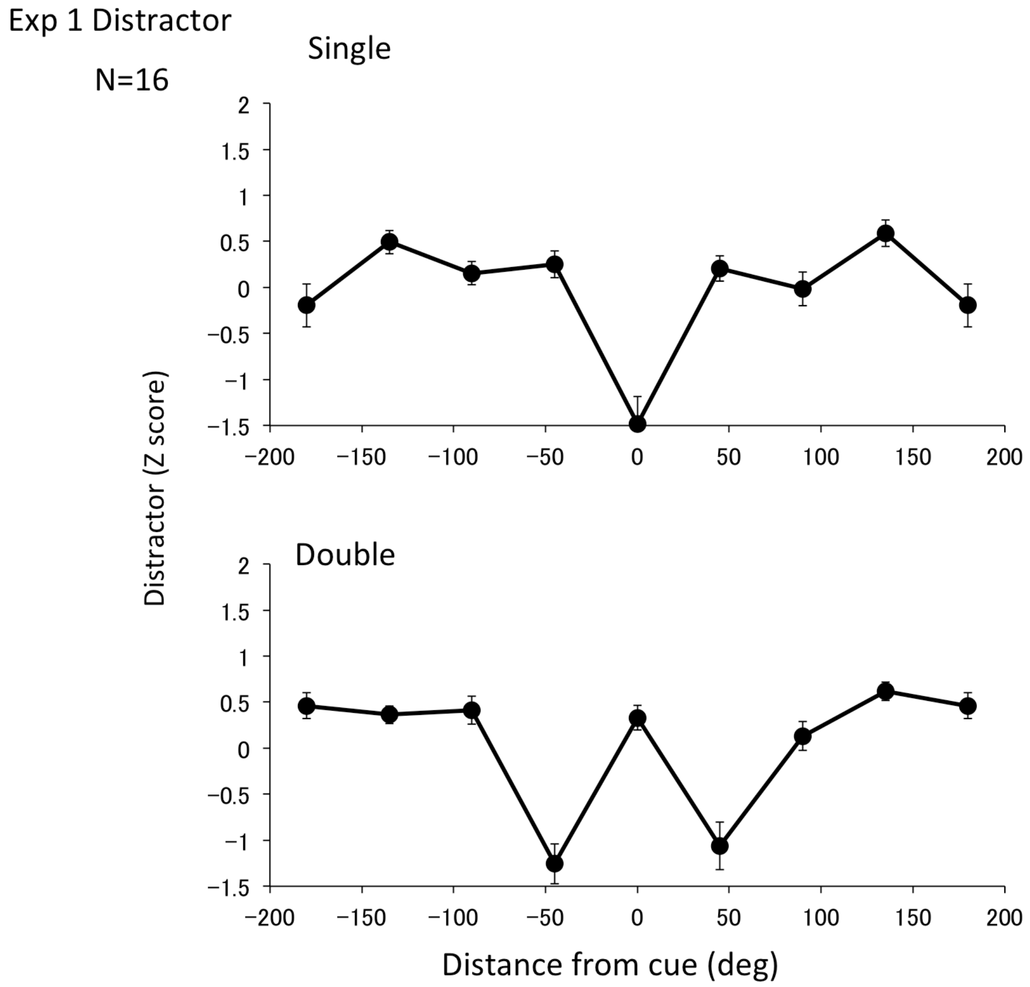

Supplementary Figure 6

P3 amplitude for distracters in the Single and Double cue conditions of Experiment 1. The average amplitude between 400 and 600 ms after distractor presentation is shown as a function of distractor presented location relative to cue. The inhibitory effect at cued locations is seen.

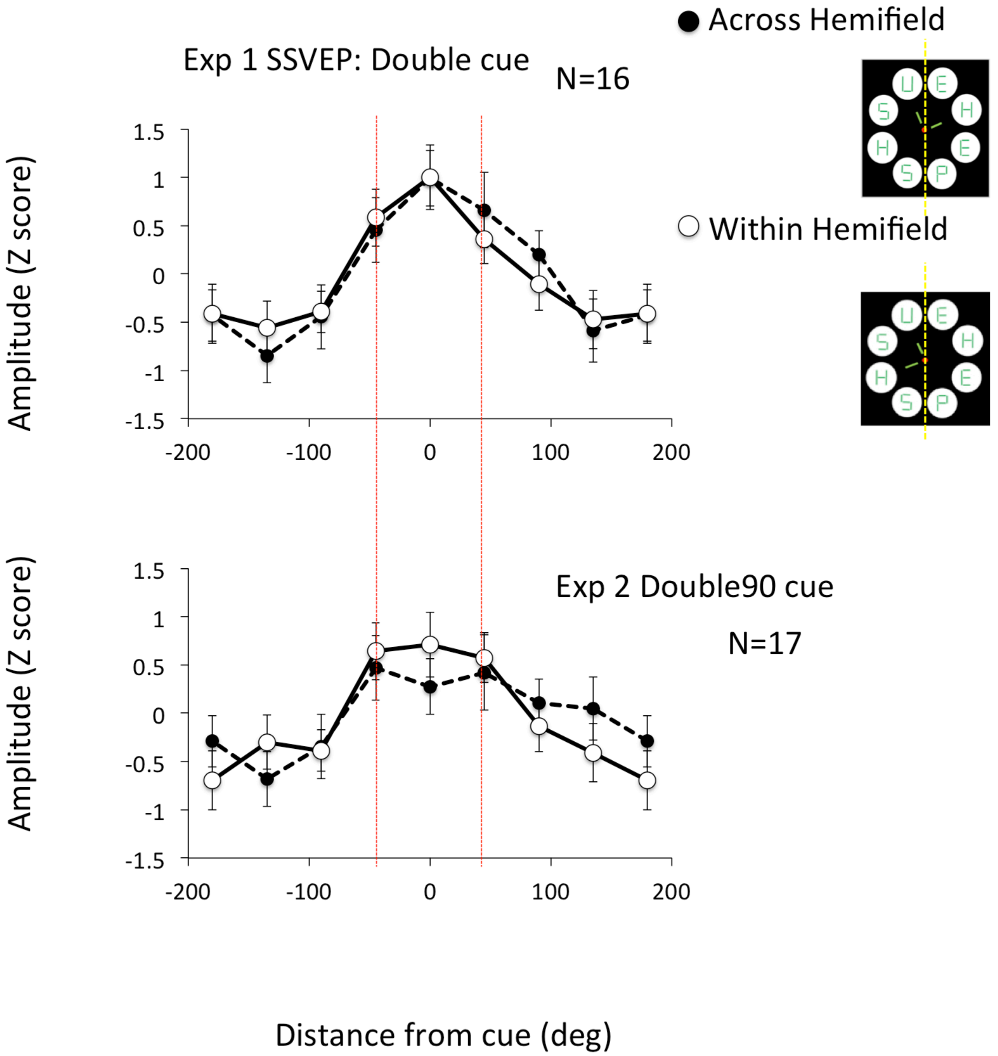

Supplementary Figure 7

SSVEP amplitude of trials with two cues in opposite hemifields (across version) and that of trials with two cues within the same hemifield (within version) of the Double (top) and Double90° (bottom) conditions of Experiments 1 and 2.

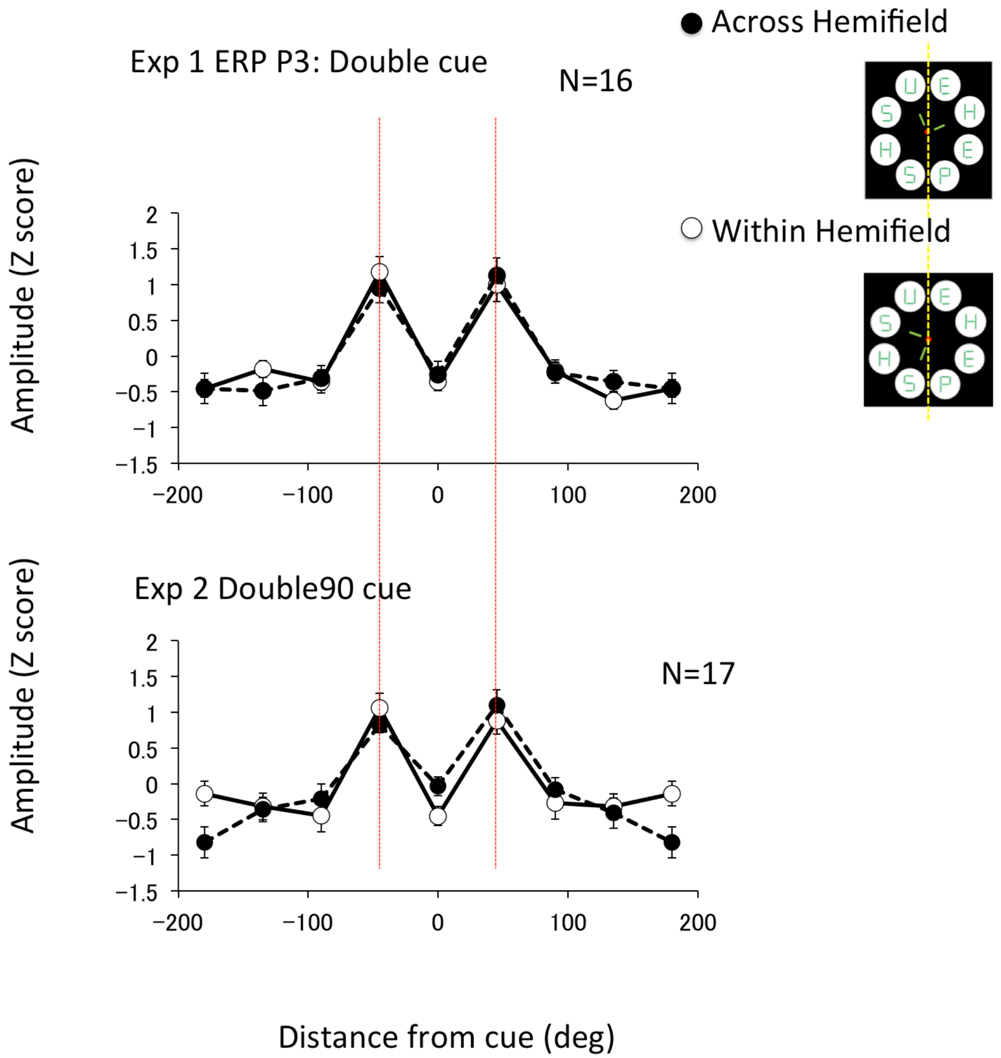

Supplementary Figure 8

P3 amplitude of trials with two cues in opposite hemifields (across version) and that of trials with two cues within the same hemifield (within version) of the Double (top) and Double90° (bottom) conditions of Experiments 1 and 2.

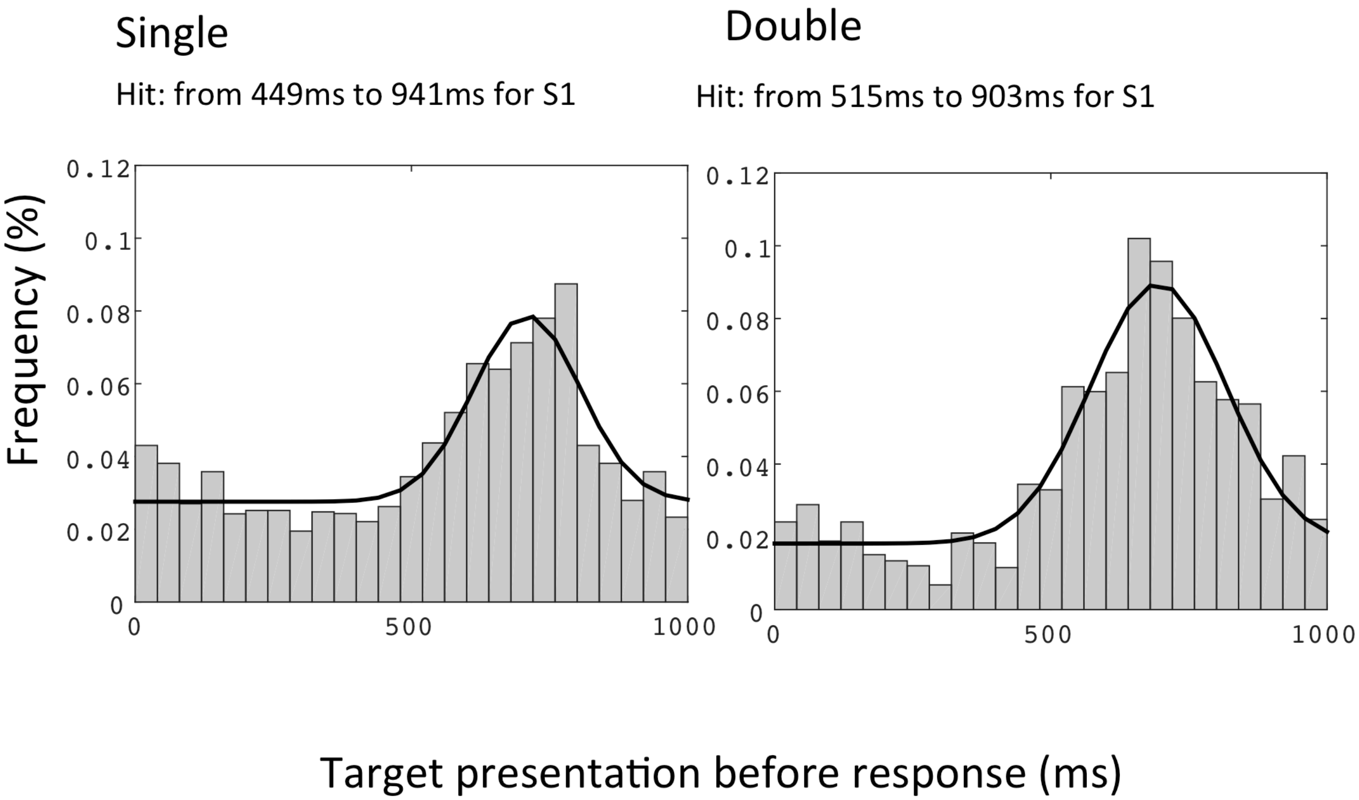

Supplementary Figure 9

Histograms of the RT to target detection. RT was defined as the time after the target presentation closest in time to each key press. To determine the time window for the response to be judged a "hit", we fitted a Gaussian function to the RT distribution. Responses within the time window $\pm 2\sigma$ of the fitted function were regarded as "hits" and the rest of the responses as "false alarms (FAs)". The FA rate was defined as the percentage of FAs against all distractors presented during a trial (or the number of response outside the hit temporal window over the total number of letter presentations (30/trial) minus the number of target presentations (7/trial on average).
